# Antioxidant and Antiproliferative Activities of Hemp Seed Proteins (*Cannabis sativa* L.), Protein Hydrolysate, and Its Fractions in Caco-2 and THP-1 Cells

**DOI:** 10.3390/ijms262311741

**Published:** 2025-12-04

**Authors:** Merit Valeria Juárez-Cruz, Cristian Jiménez-Martínez, Javier Vioque, Julio Girón-Calle, Lucía Quevedo-Corona

**Affiliations:** 1 Escuela Nacional de Ciencias Biológicas, Instituto Politécnico Nacional (IPN), Av. Wilfrido Massieu S/N, Unidad Profesional Adolfo López Mateos, Zacatenco, Alc. Gustavo A. Madero, Ciudad de México C.P. 07738, Mexico; mjuarezc1802@alumno.ipn.mx; 2 Instituto de la Grasa (C.S.I.C.), Campus Universidad Pablo Olavide, Cta de Utrera Km.1, 41013 Sevilla, Spain; jvioque@ig.csic.es (J.V.); jgiron@ig.csic.es (J.G.-C.)

**Keywords:** hemp, proteins, protein hydrolysates, Caco-2, THP-1, antioxidant, antiproliferative

## Abstract

This study evaluated the in vitro antioxidant and antiproliferative activity of hemp seed (*Cannabis sativa* L.) protein isolate, protein hydrolysate, and its fractions. The protein hydrolysate was obtained through sequential enzymatic digestion using pepsin and pancreatin, achieving a degree of hydrolysis of 48.11%. The hydrolysate was then fractionated by ultrafiltration. Assays conducted on Caco-2 (colorectal cancer) and THP-1 (leukemia) cell lines revealed that the higher-molecular-weight fraction of (>10 kDa) exhibited the strongest, concentration-dependent antiproliferative effect, as determined by the neutral red uptake (NRU) assay for Caco-2 cells and the MTT assay for THP-1 cells. Furthermore, a significant intracellular antioxidant activity was observed, particularly in the whole hydrolysate and its low-molecular-weight fractions, as measured by the DCFH-DA assay in Caco-2 cells. The results suggest the potential application of hemp seed protein hydrolysate and its fractions as antioxidant and chemoprotective supplements in oncologic therapies.

## 1. Introduction

Archaeobotanical records and plant remains from sites in Central Asia and Europe provide evidence of the use of the genus “Cannabis” since the Bronze Age, primarily for obtaining fibers and oils used in ritual activities [1,2]. The species of greatest commercial interest is *Cannabis sativa* L. (commonly known as hemp), which is phenotypically characterized by its long stem and few branches and genotypically by its minimum content (<0.3%) of delta-9-tetrahydrocannabinol (THC)—a psychoactive compound—thereby meeting established international legal limits. The hemp plant has a wide range of applications: essential oils and non-psychoactive compounds, such as cannabidiol (CBD), are extracted from its inflorescence for medical and cosmetic purposes, and the fiber from its stem is widely used in the textile and paper industries and as a biocomposite. Nutritionally, hemp seeds are noteworthy for being an excellent source of protein, polyunsaturated fatty acids (omega-3 and omega-6), dietary fiber, vitamins (especially vitamin E), and minerals such as iron, zinc, and magnesium, positioning them as a superfood for human nutrition [3].

Hemp’s protein profile is characterized by the presence of edestin (main globulin), albumin, and vicilin, which contribute to its high nutritional value. These proteins are highly digestible and possess a complete amino acid profile, thereby meeting the essential requirements for human nutrition [4].

However, to enhance their bioactivity, native proteins are often subjected to enzymatic hydrolysis, a process that facilitates the cleavage of peptide bonds, thereby producing lower-molecular-weight fractions known as bioactive peptides [5]. The efficiency of this process and the profile of the resulting peptides are influenced by experimental parameters, such as the type of enzymes used, hydrolysis time, temperature, and pH, all of which determine the degree of hydrolysis (DH). Furthermore, the intrinsic biological activity of peptides—such as antioxidant, antihypertensive, or immunomodulatory activities—depends not only on molecular weight but also on the amino acid sequence, spatial structure, and the identity of amino acid residues at the N- and C-terminal positions [6,7]. It is important to highlight that hemp proteins contain high concentrations of glutamine, arginine, and asparagine, which play critical roles in immune modulation by regulating inflammatory processes and maintaining the integrity of the intestinal epithelial barrier [8]. Hemp seed protein hydrolysates have shown diverse biological activities, including antioxidant [8,9], neuroprotective [10], antihypertensive [11], immunomodulatory, and hypoglycemic effects [8]. Nevertheless, the antiproliferative potential of protein hydrolysates against models of hematologic and solid neoplasia, specifically leukemia and colorectal cancer, remains largely unexplored. To address this gap, two representative cellular models were chosen. The Caco-2 cell line, widely used as a model of human colorectal adenocarcinoma, is notable for its spontaneous differentiation into an enterocyte-like phenotype, developing apical microvilli and tight junctions at confluence. This characteristic makes it a dual-purpose model suitable for simultaneously evaluating the cytotoxicity and intestinal absorption of bioactive peptides [12]. Additionally, the THP-1 cell line, obtained from a human myelomonocytic acute leukemia [13,14], provides a robust in vitro model for the study of hematologic neoplasia.

In this context, the present study aims to evaluate (1) the antioxidant capacity of hemp seed proteins, a protein hydrolysate, and their peptide fractions separated by molecular weights in the Caco-2 intestinal cell model, and (2) their effects on proliferative and cellular viability using colorectal cancer (Caco-2) and myelocytic leukemia (THP-1) cell models. The overall aim is to explore their potential application as functional ingredients, where their antiproliferative effects could selectively target cancer cells while their antioxidant properties help maintain systemic redox balance. This dual bioactivity is crucial for developing supportive adjuvant therapies in oncology as health-promoting strategies.

## 2. Results and Discussion

### 2.1. Composition of Defatted Flour

Table 1 presents comparative data on the nutritional composition of whole hemp seed flour (HS) and defatted hemp seed flour (HSF), specifically focusing on total protein, total fat, and moisture content. Technological processing produced significant differences between the two flours. The total protein content of the defatted flour (HSF) increased markedly to 57.84 ± 3.56%, compared with 20.25 ± 0.09% in the whole flour (HS). In contrast, the total fat content (ether extract) decreased from 57.54 ± 0.20% in HS to 0.89 ± 0.01% in HSF. Although lipid reduction improves oxidative stability, it results in the loss of beneficial omega-3 and omega-6 fatty acids [15,16]. Additionally, the higher moisture content observed in HSF (10.03 ± 0.37%) compared with HS (2.88 ± 0.43%) suggests increased hygroscopicity due to greater flour porosity, which necessitates controlled storage conditions to prevent microbiological deterioration [17]. Overall, these findings indicate that HSF is more suitable for producing high-protein food products or for obtaining concentrated protein isolates, whereas HS is preferable for applications that prioritize the inclusion of healthy lipids.

### 2.2. Determination of Protein Isolation Conditions

**Figure 1:**
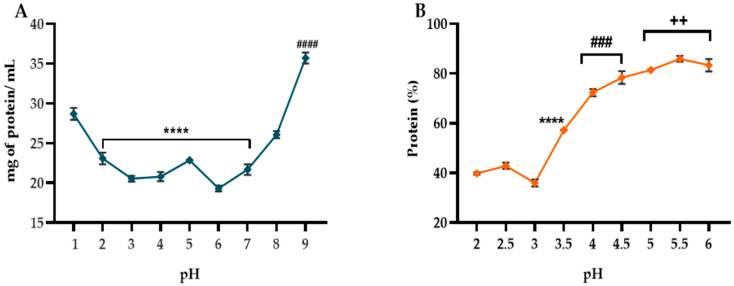
(**A**) Effect of the pH on the solubility of hemp seed proteins (mg of soluble protein/ mL); (**B**) determination of the isoelectric point (IP) of the hemp seed proteins. Data represent the average of three independent replicates ± SD. Symbols indicate statistically significant differences after one-way ANOVA and Tukey test (++ = *p* < 0.01, ### = *p* < 0.001, **** or #### = *p* < 0.0001) between the different pH treatments.

The results (Figure 1) indicate that the solubility of hemp seed protein exhibits a “U-shaped’’ pattern as a function of pH, which is characteristic of plant globular proteins. In the acidic range (pH 1–3), solubility sharply decreases (from 28.70 to 20.54 mg eq. P/mL) due to the neutralization of positive charges and continues to decline progressively until reaching the expected isoelectric point (pI ~ 5–6). The lower solubility values (19.32–22.86 mg eq. P/mL) observed between pH 4 and 7 correspond to this isoelectric region, where proteins lose electrostatic repulsion and tend to aggregate [18]. Contrarily, at alkaline pH levels (8–9), solubility increases significantly (from 26.08 to 35.72 mg eq. P/mL) because of enhanced intermolecular repulsion caused by the ionization of carboxylic and amine groups [19].

Figure 1B shows that protein precipitation is most efficient near the isoelectric point (pI), yielding 72.35–85.91%, where hydrophobic interactions are predominant [20]. In contrast, under acidic conditions, the precipitation yield decreases markedly to 35.96–42.86%. Based on this inverse relationship, pH 9 was selected as the optimal condition for protein solubilization, and pH 5.5 for precipitation. This approach produced a protein isolate with a total protein content of 87.73 ± 2.10 mg.

### 2.3. Evaluation of the Degree of Hydrolysis and the Amino Acid Profile

Bioactive peptides are short sequences of 2 to 20 amino acids encrypted within the primary structure of native proteins. These peptides are released through the hydrolysis of the precursor protein, which can be achieved using technological processing, microbiological methods, or digestive enzymes [21,22].

The degree of hydrolysis (DH) reflects the extent of peptide bond cleavage and determines the molecular weight distribution of the resulting peptides. A high DH indicates a greater proportion of low-molecular-weight peptides with shorter amino acid chains, whereas a low DH suggests the predominance of larger oligopeptides and intact proteins [10,23]. Consequently, the biological activity of peptides is intrinsically associated with their size, with those possessing a molecular weight below 1 kDa typically exhibiting the highest bioactivity. This study achieved an extensive degree of hydrolysis (DH) of 48.11 ± 0.16% for the hemp protein isolate (PI) through sequential enzymatic hydrolysis using pepsin followed by pancreatin. This value exceeds the 30% threshold typically regarded as indicative of extensive hydrolysis and substantial peptide bond cleavage [24], suggesting that the resulting hydrolysate is rich in low-molecular-weight peptides with strong potential for bioactivity. In addition to generating bioactive peptides, enzymatic hydrolysis enhances the functional properties of proteins by improving solubility across a broad pH range and reducing or eliminating allergenic epitopes [25].

The degree of hydrolysis (DH) of hemp protein is known to be highly dependent on the choice of enzymes and the specific reaction conditions. For example, Aiello et al. [26] reported that pancreatin produced the highest DH (47.5%), whereas pepsin yielded the lowest (19.7%). Notably, their high DH with pancreatin required a prolonged incubation period of 16 h, which is substantially longer than the total 3 h reaction time employed in the sequential two-enzyme system used in the present study. Sequential hydrolysis with complementary enzymes has been validated as an efficient strategy for achieving a high degree of hydrolysis (DH). This synergistic effect is demonstrated by the work of Rodriguez-Martin et al. [10], who obtained a neuroactive peptide from hemp protein. Their process involved an initial hydrolysis with the endoprotease alcalase, which yielded 30% DH, followed by treatment with the exoprotease flavourzyme, increasing the DH to 60%. This illustrates the complementary action of these enzyme classes: endoproteases hydrolyze internal peptide bonds, whereas exoproteases release small peptides and free amino acids. In the present study, this principle was applied by simulating the human gastrointestinal digestive process through sequential hydrolysis with pepsin and pancreatin [27]. The extensive hydrolysis achieved under these conditions provides a robust predictive model for the release, absorption, and bioavailability of bioactive peptides [28].

The initial hydrolysis phase employed pepsin, an aspartic endopeptidase secreted by gastric chief cells. This enzyme catalyzes the non-specific cleavage of internal peptide bonds, with a preference for hydrophobic residues such as phenylalanine, tyrosine, and tryptophan [29]. This activity primarily generates long peptides and oligopeptides while simultaneously creating new amino and carboxyl termini that serve as optimal substrates for subsequent enzymatic hydrolysis steps. The hydrolysis was then continued with pancreatin, an enzymatic extract from porcine pancreas containing trypsin, chymotrypsin, elastase, and carboxypeptidases A and B, which are naturally secreted into the duodenum [23,30]. In this phase, the endopeptidases further hydrolyze the internal bonds of the peptides produced by pepsin, while exopeptidases like carboxypeptidases act on the C-terminal ends, releasing free amino acids. The result of this extensive sequential hydrolysis is a high proportion of low-molecular-weight peptides (<1 kDa), a profile associated with enhanced biological activity and improved intestinal absorption [5].

The functional and bioactive properties of protein hydrolysates are determined not only by the selection of enzymes but also by the pretreatment conditions. Contrary to expectations, Vu et al. [31] demonstrated that preheating sorghum proteins to 100 °C for 15–30 min significantly reduced the degree of hydrolysis (DH) compared to unheated controls. This suggests that thermal denaturation induced the formation of aggregated structures, shielding proteolytic cleavage sites from enzyme access [32].

The bioactivity of the resulting hydrolysates and peptide fractions is governed by their molecular weight distribution and amino acid composition [33,34]. For instance, a high content of hydrophobic amino acids is a key determinant of antioxidant capacity, as their aromatic groups can donate protons to neutralize free radicals and quench reactive oxygen species generated during lipid peroxidation [22,35].

To isolate peptides with distinct bioactivities, the hemp seed protein hydrolysate (HSH) was fractionated by cascade tangential ultrafiltration using membranes with molecular weight cut-offs of 10, 5, and 1 kDa. This process produced four fractions based on molecular weight: F1 (>10 kDa), F2 (10–5 kDa), F3 (5–1 kDa), and F4 (<1 kDa). The amino acid profiles of the native protein isolate (PI), the total hydrolysate (HSH), and each fraction were quantified using high-performance liquid chromatography (HPLC) following acid hydrolysis.

As shown in Table 2, the enzymatic hydrolysis process preserved the overall amino acid composition of the protein isolate (PI), with no significant loss of essential amino acids. Glutamate/glutamine, aspartate/asparagine, arginine, and leucine were the predominant amino acids across all samples, consistent with previously reported profiles of hemp seed proteins [11,22]. Notably, all samples were enriched in functional amino acids associated with immunomodulatory activity, particularly arginine, glutamine, and histidine. Arginine serves as a precursor for nitric oxide. It plays a crucial role in lymphocyte proliferation, whereas glutamine functions as an essential energy source for immune cells such as macrophages and lymphocytes [9,10]. Consequently, the relative enrichment of these amino acids in the lower-molecular-weight fractions, especially F4 (<1 kDa), may indicate their enhanced potential to stimulate immune responses [36].

Analysis of the molecular weight fractions revealed a divergent amino acid profile compared to the original hydrolysate (HSH). A comparative increase in serine was consistent across all fractions. Notably, the F1 fraction (>10 kDa) exhibited an elevated glycine content but a reduced proline concentration, implying an enrichment of proline in the F4 fraction (<1 kDa). This redistribution is particularly relevant as proline has been identified as a contributor to antioxidant capacity [4].

### 2.4. Antiproliferative Effect of PI, HSH, and Fractions

**Figure 2:**
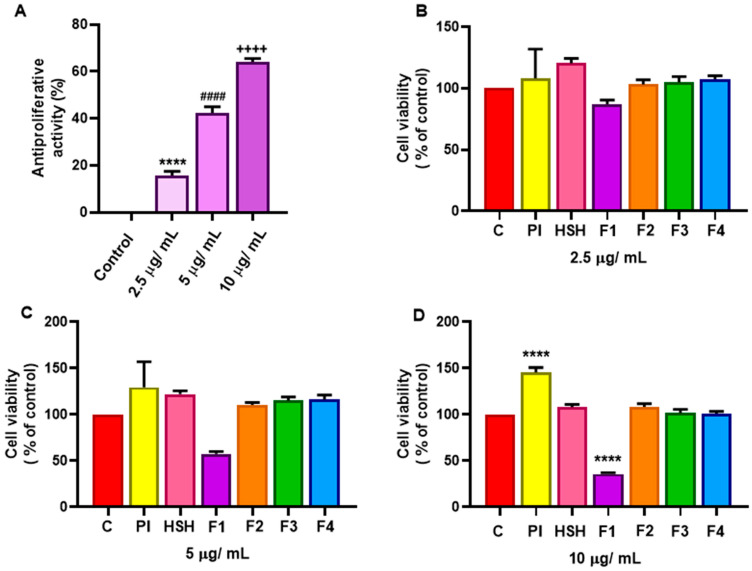
Effect of protein isolate (PI), hydrolysate (HSH) and its fractions [F1 (>10 kDa), F2 (5–10 kDa), F3 (1–5 kDa) and F4 (<1 kDa)] at different concentrations: 2.5 μg/ mL, 5 μg/ mL and 10 μg/ mL, on cell viability of THP-1 cells: (**A**) expressed as antiproliferative activity of the fraction F1 at different concentrations; (**B**–**D**) expressed as cell viability at the same concentrations. Cells (1.2 × 10^4^ cells/well) were cultured in 96-well plates in culture medium containing different concentrations. Cell number was estimated using the MTT assay. Data represent media ± standard error of six incubations. Symbols indicate statistically significant differences between groups (experimental and control cells grown without the addition of protein or peptides) after one-way ANOVA and Tukey test (**** or #### or ++++ = *p* < 0.0001).

To evaluate the antiproliferative effect of PI, HSH, and derived fractions from hemp seed proteins, the cell lines Caco-2 and THP-1were used as in vitro models of cancer. Figure 2 shows the results of the THP-1 line, a model derived from the peripheral blood of a patient with acute monocytic leukemia. This cell line, which grows in suspension, is well-established for studying myeloid leukemia and immunomodulation [13,14].

Following treatment with concentrations of 2.5, 5, and 10 µg/mL, on the fourth day, a significant dose-dependent reduction in cell viability (Figure 2B), which we also expressed as an antiproliferative effect (Figure 2A), was observed exclusively with the F1 fraction (>10 kDa) in THP-1 cells. At the highest dose of 10 µg/mL, this fraction achieved 60% inhibition of proliferation. Based on the applied MTT and neutral red uptake assays, which measure metabolic activity and lysosomal integrity, respectively, we cannot definitively discriminate between a reduction in cell viability (growth-inhibiting) and a cytotoxic (cell-killing) effect. However, the observed concentration-dependence and the sustained inhibition over five days are more characteristic of a cytostatic mechanism. We therefore hypothesize that the higher molecular weight bioactive peptides in F1 can interfere with cell cycle mechanisms, a phenomenon previously documented with other bioactive peptides in leukemia models [37,38]. The lack of activity in the different fractions indicates that this biological effect is specific to the unique peptide composition of F1.

The selective antiproliferative activity is potentially driven by a unique amino acid composition rich in glutamine, histidine, and arginine. Beyond their shared role as precursors for immune function [10,39], arginine possesses a specific, documented capacity to inhibit proliferation and induce apoptosis in leukemia cells. This occurs through the modulation of nitric oxide synthesis and key enzymatic pathways [40,41], which constitutes a plausible mechanism underlying the results reported here.

In contrast, Caco-2 cells serve as a standard model for colorectal cancer [42]. These cells actively proliferate, forming a confluent and polarized monolayer after approximately 14 days in culture. Upon confluence, they undergo phenotypic differentiation into enterocyte-like cells, characterized by well-developed microvilli and functional tight junctions [43,44]. These unique characteristics make them suitable for assessing both general cytotoxicity and specific effects on intestinal barrier integrity and function.

**Figure 3:**
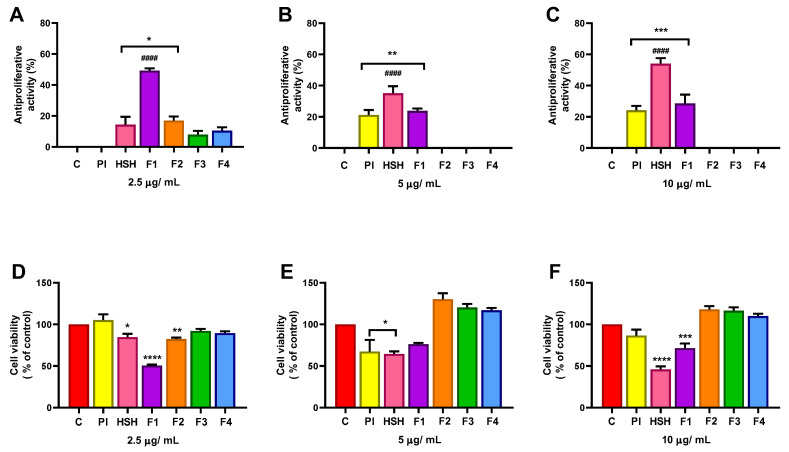
Effect of the protein isolate (PI), hydrolysate (HSH) and its fractions [F1 (>10 kDa), F2 (5–10 kDa), F3 (1–5 kDa) and F4 (<1 kDa)] at different concentrations: (**A**) 2.5 μg/ mL, (**B**) 5 μg/ mL, and (**C**) 10 μg/ mL, on proliferation of Caco-2 cells expressed as antiproliferative activity; (**D**–**F**) cell viability at the same concentrations. Cells (1.4 × 10^4^ cells/well) were cultured in 96-well plates in culture medium containing different concentrations. The cell number was estimated by determining the neutral red uptake. The data represent the average of six incubations ± standard error. Asterisks indicate significant differences when compared to the control group (cells grown without the addition of protein or peptides) (* = *p* < 0.05, ** = *p* < 0.01, *** = *p* < 0.001, and **** = *p* < 0.0001); #### = *p* < 0.0001 indicates significant differences between all other groups.

When exposed to the same conditions and concentrations (2.5, 5, and 10 µg/mL) as the THP-1 cells, the Caco-2 cells demonstrated greater susceptibility. All fractions induced inhibitory effects, even at the lowest concentration (Figure 3). The highest reduction in cell viability was observed for the F1 fraction (>10 kDa) at 2.5 µg/mL and for the HSH at 5 and 10 µg/mL. In all effective cases, treatment induced more than 35% inhibition compared to the control. This differential response suggests a cell-type-specific mechanism of action, potentially mediated by variations in membrane receptor expression, cellular permeability, or intracellular signaling pathways in epithelial cells [44].

**Figure 4:**
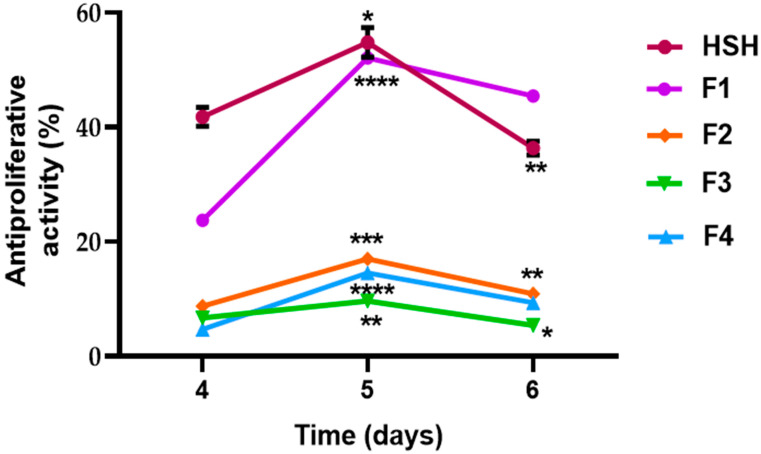
Hemp seed protein hydrolysate (HSH) and its fractions [F1 (> 10 kDa 2.5 μg/mL), F2 (5–10 kDa 5 μg/mL), F3 (1–5 kDa 2.5 μg/mL), and F4 (< 1 kDa 2.5 μg/mL)] on the proliferation of Caco-2 cells. Cells (1.4 × 10^4^ cells/well) were cultured in 96-well plates in culture medium containing different concentrations. The cell number was estimated by determining the neutral red uptake. The data represent the average of six incubations ± standard error. Asterisks indicate significant difference between samples at different days of the two-way ANOVA and Tukey test (* = *p* < 0.05, ** = *p* < 0.01, *** = *p* < 0.001, and ****= *p* < 0.0001).

The prolonged inhibition of cellular proliferation was evaluated in a separate experiment, wherein cells were incubated for up to 4 days with the most bioactive fractions (Figure 4). The results confirmed a sustained reduction in cell viability. Continuous treatment with HSH (10 µg/mL) and the F1 fraction (>10 kDa, 2.5 µg/mL) maintained a significant inhibition of proliferation—exceeding 20%—throughout the study period. The persistence of this effect suggests that the active compounds not only cause acute inhibition but may also induce prolonged cell cycle arrest or trigger apoptotic pathways. This hypothesis, however, requires future validation through techniques such as flow cytometry [45].

This work aligns with the growing research focus on the anticancer potential of bioactive peptides. For instance, Wei et al. [45] demonstrated that a hemp seed protein hydrolysate induced apoptosis in human hepatocarcinoma cells (Hep3B) and reduced their viability and proliferation by 50% at a concentration of 10 mg/mL after 24 h. Critically, this cytotoxic effect was selective, as it was not observed in non-cancerous hepatocytes (L02), indicating a preferential mechanism of action against transformed cells.

Furthermore, Givonetti et al. [46] investigated the effects of hemp protein and its hydrolysate on human myeloid leukemia (U937) cells and revealed a functional dichotomy between the two forms. The intact (native) protein exhibited greater potency in inhibiting U937 cell proliferation, implying that its intrinsic peptide sequence retains specific bioactive conformations that may be disrupted by hydrolysis. In contrast, the hydrolysate displayed enhanced antioxidant capacity. This distinct biofunctional profile positions the native protein and its hydrolysate as complementary therapeutic candidates with different potential applications—the hydrolysate’s pronounced antioxidant activity may be particularly valuable in mitigating oxidative stress associated with chronic inflammatory conditions such as inflammatory bowel disease. The observed antiproliferative effects align with findings from other plant-derived peptides. For instance, Fan et al. [47] reported that quinoa protein hydrolysates (<5 kDa) potently inhibited Caco-2 cell proliferation by over 50%. They attributed this effect to the inhibition of histone deacetylase 1 (HDAC1) and the NF-κB pathway, coupled with the activation of caspase-3, a key mechanism in apoptosis and tumor suppression. The similarity in activity suggests that hemp seed peptides may share a comparable mechanism, acting as epigenetic modulators and inducers of apoptosis in gastrointestinal cancer cells.

### 2.5. Effect on Antioxidant Capacity in the Caco-2 Cell Line

Reactive oxygen species (ROS), such as the superoxide anion (O_2_•^−^), hydrogen peroxide (H_2_O_2_), and hydroxyl radical (•OH), are highly reactive and unstable oxygen-derived molecules. Under physiological conditions, they are primarily generated as byproducts of mitochondrial metabolism and by specific enzymic systems like NADPH oxidases (NOX), which play essential roles in host defense [48]. However, when the redox equilibrium shifts toward oxidation, a state of oxidative stress arises. This imbalance leads to extensive damage to cellular macromolecules through lipid peroxidation, protein carbonylation, and DNA lesions—processes that underpin the pathophysiology of numerous disorders, including cancer, neurodegenerative diseases, and metabolic conditions such as diabetes mellitus [49,50]. Consequently, dietary supplementation with antioxidant compounds represents a promising therapeutic approach to counteract ROS overproduction, restore redox homeostasis, and reinforce endogenous defense mechanisms [49].

The intracellular antioxidant capacity of the protein isolate (PI), hydrolysate (HSH), and their fractions were evaluated in Caco-2 cells using the 2′,7′-dichlorofluorescein-diacetate (DCFH-DA) assay. In this method, the cell-permeant DCFH-DA probe diffuses into cells, where intracellular esterases hydrolyze it to the non-fluorescent 2′,7′-dichlorofluorescein (DCFH), which is trapped within the cytosol [51]. Upon exposure to oxidizing species, DCFH is irreversibly oxidized to the highly fluorescent dichlorofluorescein (DCF). To induce oxidative stress, ABAP was added to the incubation medium [52]. Since the resulting fluorescence intensity is directly proportional to intracellular ROS levels, a lower signal indicates a higher antioxidant capacity of the samples. This can result from either the direct scavenging of ABAP-generated ROS or the activation of cellular antioxidant mechanisms [53]. Thus, this assay effectively differentiates the ability of peptides to attenuate oxidative stress in a cellular model.

**Figure 5:**
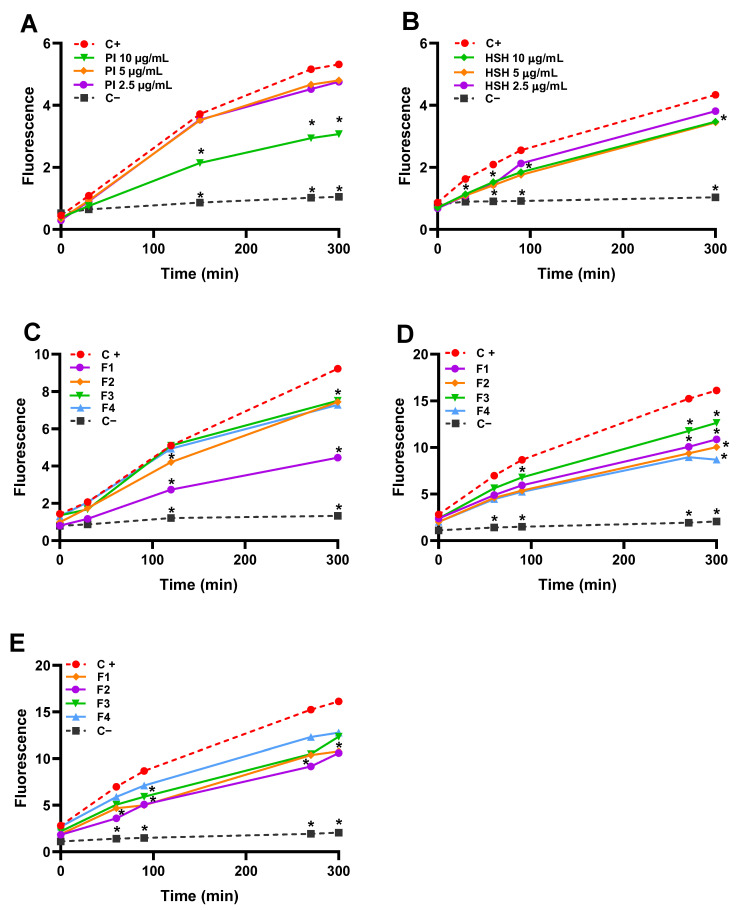
Effect of the protein isolate (PI), hemp seed protein hydrolyzed (HSH) and its fractions [F1 (>10 kDa), F2 (5–10 kDa), F3 (1–5 kDa), and F4 (<1 kDa)] in different concentrations: (**A**) PI, (**B**) HSH, (**C**) 2.5 μg/ mL, (**D**) 5 μg/ mL, and (**E**) 10 μg/ mL of protein/ mL in the inhibition of the DCFH oxidation in Caco-2 cells. C− = negative control (cells without both PI and ABAP), C+ = positive control (cells with ABAP but without samples). Data represent the average of six incubations ± standard error; error bars are not shown for clarity. Asterisks indicate a significant difference between the C+ and the different samples (two-way ANOVA and Tukey test, * = *p* < 0.05).

As shown in Figure 5A, the protein isolate (PI) at a concentration of 10 µg/mL exhibited the strongest intracellular antioxidant activity, significantly lowering ROS levels compared with the ABAP-treated positive control (*p* < 0.05). This indicates that the native protein structure itself may confer protective capacity at this specific concentration. Conversely, the Hydrolysate (HSH) displayed a clear dose-dependent response, with the 10 µg/mL concentration again producing the most pronounced reduction in ROS. This concentration differed significantly from the positive control and demonstrated greater ROS inhibition than the lower concentrations evaluated (5 and 2.5 µg/mL). The superior efficacy of HSH is consistent with previous reports attributing the significant antioxidant activity of enzymatic hydrolysates to the exposure of hydrophobic amino acid residues and the release of bioactive peptides capable of donating electrons to neutralize free radicals [6,34].

Analysis of the HSH fractions revealed that antioxidant activity was influenced by an interaction between molecular weight and concentration (Figure 5C–E). At the highest concentration evaluated, only the high-molecular-weight fraction (>10 kDa) exhibited significant activity, possibly due to the formation of peptide aggregates or specific structural conformations capable of effectively trapping free radicals. In contrast, at lower concentrations (2.5 and 5 µg/mL), all fractions displayed significant antioxidant activity, with the >10 kDa fraction being most effective at 2.5 µg/mL and the <1 kDa fraction at 5 µg/mL. This duality indicates distinct modes of action: high-molecular-weight peptides may act with high specificity at low doses, whereas smaller peptides (<1 kDa) benefit from enhanced bioavailability and cellular uptake, enabling efficient intracellular radical scavenging in a dose-dependent manner [54,55]. The underlying mechanisms—such as direct ROS neutralization, metal ion chelation, and modulation of signaling pathways governing antioxidant enzyme expression, inflammation, and apoptosis [55]—remain to be elucidated and represent a primary objective for future research.

The antioxidant potential of protein hydrolysates is well-established, as exemplified by a recent study on hemp. Gao et al. [56] demonstrated that hemp protein hydrolysates at a concentration of 0.4 mg/mL exhibited strong in vitro antioxidant properties, achieving 52% ABTS^+^ radical scavenging and 52.9% metal chelation activity. In a cellular model, these hydrolysates significantly mitigated H_2_O_2_-induced oxidative stress in HepG2 cells by increasing cell viability from 55.7% to 80%, reducing oxidative biomarkers, and enhancing the enzymatic activities of superoxide dismutase (SOD), catalase (CAT), and glutathione peroxidase (GPx). Such bioactivity is associated with peptide structural characteristics, as the amino acid sequence, composition, and molecular weight are major determinants of antioxidant efficacy [7]. In particular, the presence of hydrophobic and aromatic amino acids facilitates electron donation, radical stabilization, and interactions with cell membranes [8,22]. Given that these key amino acids are abundant in both the HSH and its ultrafiltration fractions, they underpin the antioxidant effects observed in the present cellular model.

## 3. Materials and Methods

### 3.1. Biological Materials and Reactive

Hemp seeds were sourced from OKKO Super Foods. The enzymes pepsin (Sigma,. P7125) and pancreatin (Sigma, P3292), along with ABAP (2,2′-azobis(2-amidinopropane) dihydrochloride), DCFH-DA (2′,7′-dichlorofluorescein diacetate), and BCA (bicinchoninic acid) reagents, were purchased from Sigma-Aldrich (St. Louis, MO, USA). For cell culture, Dulbecco’s Modified Eagle Medium (DMEM), Hank’s Balanced Salt Solution (HBSS), and fetal bovine serum (FBS) were obtained from GIBCO (Barcelona, Spain). All other reagents used were of analytical grade and procured from J.T. Baker (Phillipsburg, NJ, USA), Merck (Darmstadt, Germany), and Bio-Rad Laboratories, Inc. (Hercules, CA, USA). The Caco-2 and THP-1 cell lines were kindly provided by the Laboratory of Bioactive and Functional Components of Plant Products, Instituto de la Grasa (Seville, Spain).

### 3.2. Obtention of the Hemp Defatted Flour

Hemp seed defatted flour was prepared following the method of Tian et al. [57]. with slight modifications. The seeds were finely ground, followed by a defatting process consisting of five ultrasound-assisted extraction cycles of 60 min each, using 100% *n*-hexane as the solvent at a 1:5 (*w*/*v*) ratio and a frequency of 60 kHz. After each cycle, the solvent was removed and replaced with fresh *n*-hexane. To eliminate residual phenolic compounds, the defatted material was treated twice with 100% acetone at a 1:10 (*w*/*v*) ratio under continuous stirring for one hour per extraction. The mixture was decanted after each step, and the resulting flour was air-dried under a fume hood to remove solvent traces. The final product, free of lipids and phenolic compounds, was milled to obtain the hemp seed flour (HSF).

### 3.3. Preparation of Hemp Seed Protein Isolate (PI)

To isolate the protein, its solubility curve was initially determined. For this purpose, HSF was suspended in distilled water (1:20 *w*/*v*). The suspension was maintained under constant stirring at 37 °C for 2 h, and the pH was adjusted to various values ranging from 1 to 9. The samples were centrifuged at 4 °C for 40 min. The supernatant obtained from each pH point was collected and analyzed for soluble protein content using the bicinchoninic acid (BCA) method [58].

Subsequently, the precipitation curve was established using the supernatant obtained at the pH corresponding to maximum protein solubility. The pH was adjusted to values between 2.5 and 6.0 in 0.5-unit increments, and each sample was maintained under constant stirring for 1 h. Following this, samples were centrifuged at 8500 rpm for 90 min, and the resulting protein pellet was collected and lyophilized. The total protein concentration was quantified by the Kjeldahl method [59]. In both solubilization and precipitation steps, the pH was adjusted using 1 N NaOH or 1 N HCl.

### 3.4. Hydrolysis of Hemp Seed Protein Isolate

Enzymatic hydrolysis of the protein isolate (PI) was carried out sequentially using pepsin and pancreatin under constant stirring. The PI was suspended in distilled water at a concentration of 5% (*w*/*v*). Afterward, pepsin was added at a concentration of 4 USP/g of protein, and the mixture was incubated at pH 2.0 and 37 °C for 1 h. Subsequently, pancreatin was added at the same enzyme concentration (4 USP/g), and the pH was adjusted to 7.5 for a further 2 h of incubation at 37 °C. Enzymatic activity was terminated by heating the mixture at 80 °C for 15 min [23]. The resulting hydrolysate was centrifuged, and the supernatant was collected and stored at −80 °C before lyophilization.

### 3.5. Degree of Hydrolysis (DH)

The degree of hydrolysis (DH), defined as the percentage of peptide bonds cleaved relative to a completely hydrolyzed reference sample, was quantified using the 2,4,6-trinitrobenzenesulfonic acid (TNBS) method reported by Adler-Nissen [60]. Free amino groups were determined spectrophotometrically (340 nm) using a glycine calibration curve (0–1.6 mM) as a standard [61]. For complete hydrolysis, an aliquot of the sample was treated with 12 N HCl and incubated at 110 °C for 24 h. The degree of hydrolysis was calculated as follows:

$$\%DH=\left(\frac{free\;amino\;groups\;without\;HCl}{free\;amino\;groups\;with\;HCl}\right)\times 100$$

### 3.6. Fractionation of the Hydrolysate by Membrane Ultrafiltration

The hydrolysate was fractionated by cascade tangential flow ultrafiltration using a series of regenerated cellulose membranes in an Amicon stirred cell (Model 8400, Millipore, Billerica, MA, USA). A 10% (*w*/*v*) solution of the hemp seed protein hydrolysate (HSH) was prepared in distilled water. The solution was first filtered through a membrane with a 10 kDa molecular weight cut-off (MWCO). The retentate from this step was collected as the F1 fraction (>10 kDa). The permeate was then subsequently passed through a 5 kDa MWCO membrane; the retentate from this step was collected as the F2 fraction (5–10 kDa). This process was repeated with a 1 kDa MWCO membrane, yielding the F3 (1–5 kDa) fraction as the retentate and the F4 (<1 kDa) fraction as the final permeate. All filtration steps were performed under a constant nitrogen pressure of 50 psi at 4 °C to minimize peptide degradation [61]. The obtained fractions (F1–F4) were then freeze-dried and stored at −20 °C for subsequent analysis.

### 3.7. Amino Acid Composition Analysis

The amino acid profiles of the samples (isolate, hydrolysate, and fractions) were determined in duplicate by high-performance liquid chromatography (HPCL), following the method described by Alaiz et al. [62]. Briefly, the samples were hydrolyzed with HCL 6.0 M under a nitrogen atmosphere at 110 °C for 24 h, using D,L-α-aminobutyric acid as the internal standard. Tryptophan was quantified through alkaline hydrolysis, according to the procedure of Yust et al. [63].

### 3.8. Cell Culture

The antiproliferative activity and cell viability were evaluated in the cellular lines THP-1 and Caco-2. Both cell lines were maintained at 37 °C in a humidified atmosphere containing 5% CO_2_. Cells were cultured in Dulbecco’s Modified Eagle Medium (DMEM) supplemented with 10% (*v*/*v*) fetal bovine serum, 1% (*v*/*v*) non-essential amino acids, 100 U/mL penicillin, and 100 µg/mL streptomycin [64]. Three concentrations of protein, hydrolysates, and fractions (2.5, 5, and 10 μg/mL) were used for the analysis. The samples were suspended in distilled water and sterilized using Q-Max^®^ 0.22 μm PES (polyether sulfone) filters (MIKROLAB—FRISENETTE A/S, Viby, Denmark). Different concentrations were prepared by diluting the sterilized samples with sterile DMEM.

### 3.9. Proliferative Activity Assay in Caco-2 Cell Line

Cells were seeded in 96-well microplates at a density of 1.4 × 10^4^ cells per well in 50 µL of medium. Subsequently, 50 µL of each sample, diluted in DMEM to the desired concentrations, was added, and the plates were incubated for 4 days. After incubation, the culture medium was removed, and 50 µL of a 50 µg/mL neutral red (NR) solution was added to each well, followed by a 30 min incubation period. The NR solution was then discarded, and the wells were washed twice with Hank’s Balanced Salt Solution (HBSS). To solubilize the dye, 75 µL of 1% (*v*/*v*) acetic acid in 50% (*v*/*v*) ethanol was added. Absorbance was measured at 540 nm using a microplate reader [57,65]. Reduced cell viability was expressed as a decrease in NR uptake compared with untreated control cells.

### 3.10. Proliferative Activity Assay in THP-1 Cell Line

To evaluate cell viability through mitochondrial functional integrity, an MTT assay was performed [42,65]. The cells were seeded in a 96-well microplate at a cellular density of 1.2 × 10^4^ cells/well in 50 µL. Subsequently, 50 µL of the samples at various concentrations were added to each well, and the plates were incubated for 4 days. After incubation, 3-(4,5-dimethylthiazol-2-yl)-2,5-diphenyltetrazolium bromide (MTT) was added at a final concentration of 0.5 mg/mL in serum-free medium and incubated for 60 min. During this period, metabolically active cells reduced MTT to insoluble formazan crystals. To dissolve the formazan, 100 µL of 0.1 N HCl in isopropanol was added to each well. Following complete homogenization, absorbance was measured at 570 nm with background correction at 630 nm using a microplate reader.

### 3.11. Antioxidant Activity Assay in Caco-2 Cell Line

Antioxidant activity was assessed using a fluorometric assay based on 2′,7′-dichlorofluorescein diacetate (DCFH-DA) and the peroxidation initiator 2,2′-azobis(2-amidinopropane) dihydrochloride (ABAP), allowing for the total quantification of intracellular reactive oxygen species (ROS). In this method, higher fluorescence intensity is proportional to higher ROS concentrations [53].

For the assay, Caco-2 cells were seeded in a 96-well microplate at a density of 10^4^ cells/well in DMEM medium and cultured for 14 days under standard conditions (37 °C, 5% CO_2_, and 95% relative humidity) to achieve differentiation. Twenty-four hours prior to the experiment, the cells were treated with samples (protein, hydrolysate, or protein fractions) that had been previously sterilized and diluted in DMEM to concentrations of 2.5, 5, and 10 µg/well, and then incubated for 24 h.

Subsequently, 100 µL of a DCFH-DA solution (50 µM, 0.25% MeOH in DMEM) was added and incubated for 1 h. After this time, the medium was discarded, and the wells were carefully washed twice with 100 µL of HBSS to preserve the cell monolayer. Immediately, 100 µL of ABAP (50 µM in HBSS) was added to initiate the generation of free radicals.

The emitted fluorescence was measured at 0 time and every 30 min for 5 h with a microplate reader (Fluoroskan Ascent FL, Thermo Scientific, MA, USA) at excitation and emission wavelengths of 485 and 555 nm, respectively. The following controls were included: a positive control (C^+^) consisting of cells treated with ABAP but without sample addition, and a negative control (C^−^) consisting of cells neither treated with ABAP nor exposed to samples, with Hank’s Balanced Salt Solution (HBSS) used as a substitute.

### 3.12. Statistical Analysis

Statistical analysis was performed using GraphPad Prism 8.0 software (Boston, MA, USA). The results are expressed as the mean ± standard error or standard deviation. One-way and two-way analyses of variance (ANOVA) were performed, as well as Tukey’s comparison of means between or within groups. In all cases, *p* <0.05 was considered as the level of significance.

## 4. Conclusions

This study establishes that hemp seed protein hydrolysate (HSH) and its ultrafiltration fractions possess significant and dual biological activities. The principal finding is the identification of a potent, dose-dependent, and selective antiproliferative effect against colorectal adenocarcinoma (Caco-2) and monocytic leukemia (THP-1) cell lines, with the high-molecular-weight fraction (F1 > 10 kDa) demonstrating the greatest efficacy. Furthermore, the samples exhibited notable intracellular antioxidant activity, which was governed by a clear interaction between peptide molecular weight and concentration. The protein isolate (PI) and the complete hydrolysate (HSH) showed strong effects, and among the fractions, the high-molecular-weight F1 was most effective at a low concentration (2.5 µg/mL) while the low-molecular-weight fraction (F4, <1 kDa) was most effective at a higher concentration (5 µg/mL). Collectively, these findings underscore the dual potential of hemp seed peptides as a reduction in cell viability agents and potent antioxidants, positioning them as promising candidates for development as functional food ingredients for chemoprevention and as adjuvants in oncological therapies.

## Figures and Tables

**Table 1:** Composition of protein, fat, and moisture in the whole hemp seed flour (HS) and the defatted flour (HSF) (g/100 g dry base).

Determination	HS	HSF
Total protein (N × 6.25)	20.25 ± 0.09	57.84 ± 3.56
Fat	57.54 ± 0.20	0.89 ± 0.01
Moisture	2.88 ± 0.43	10.03 ± 0.37

The results represent the mean of three independent determinations ± standard deviation.

**Table 2:** Amino acid profiles of defatted hemp flour, hemp protein isolate, hydrolysate, and its fractions (F1–F4).

			HSH			
Amino Acids	PI	HSH	F1	F2	F3	F4
Aspartic acid ^a^	11.68 ± 0.01	8.04 ± 0.01	10.08 ± 0.00	8.92 ± 0.01	10.45 ± 0.03	6.03 ± 0.01
Glutamic acid ^b^	17.93 ± 0.06	12.44 ± 0.049	18 ± 0.01	16.86 ± 0.01	16.13 ± 0.01	10.40 ± 0.01
Serine	5.65 ± 0.01	3.40 ± 0.03	6.88 ± 0.05	6.02 ± 0.01	6.20 ± 0.01	5.41 ± 0.01
Histidine	3.07 ± 0.03	2.23 ± 0.09	3.73 ± 0.12	3.65 ± 0.09	3.40 ± 0.07	3.97 ± 0.10
Glycine	4.99 ± 0.11	3.71 ± 0.28	8.09 ± 0.03	5.56 ± 0.01	5.79 ± 0.00	4.35 ± 0.01
Threonine	3.75 ± 0.02	2.69 ± 0.01	4.90 ± 0.04	3.74 ± 0.05	4.06 ± 0.08	3.39 ± 0.01
Arginine	13.52 ± 0.13	9.04 ± 0.05	15.24 ± 0.05	14.28 ± 0.01	13.80 ± 0.00	14.14 ± 0.03
Alanine	4.61 ± 0.06	3.39 ± 0.17	3.89 ± 0.17	4.79 ± 0.00	5.21 ± 0.02	5.22 ± 0.00
Proline	1.72 ± 0.04	1.27 ± 0.07	0.90 ± 0.01	1.95 ± 0.03	1.86 ± 0.02	2.06 ± 0.08
Tyrosine	2.73 ± 0.00	2.05 ± 0.00	1.67 ± 0.01	2.79 ± 0.01	2.57 ± 0.08	4.38 ± 0.01
Valine	5.80 ± 0.04	3.93 ± 0.26	5.61 ± 0.01	5.8 ± 0.01	6.26 ± 0.40	6.66 ± 0.01
Methionine	1.17 ± 0.00	1.86 ± 0.03	1.62 ± 0.03	2.60 ± 0.02	1.88 ± 0.09	2.19 ± 0.00
Cysteine	0.03 ± 0.01	0.71 ± 0.02	0.04 ± 0.01	0.69 ± 0.02	0.02 ± 0.00	0.06 ± 0.00
Isoleucine	4.73 ± 0.03	3.32 ± 0.09	4.76 ± 0.01	4.80 ± 0.02	5.16 ± 0.02	5.00 ± 0.01
Tryptophan	1.4 ± 0.06	0.99 ± 0.03	1.2 ± 0.01	1.39 ± 0.01	1.21 ± 0.02	1.25 ± 0.04
Leucine	7.77 ± 0.03	5.26 ± 0.06	5.96 ± 0.01	7.16 ± 0.01	6.90 ± 0.01	11.22 ± 0.04
Phenylalanine	5.49 ± 0.02	3.71 ± 0.14	3.86 ± 0.01	4.75 ± 0.05	4.57 ± 0.00	9.87 ± 0.04
Lysine	3.90 ± 0.01	2.94 ± 0.02	3.61 ± 0.01	4.30 ± 0.01	4.58 ± 0.01	4.44 ± 0.01

PI = protein isolate, HSH = defatted hemp flour, fractions from the hydrolyzate: F1 (>10 kDa), F2 (5–10 kDa), F3 (1–5 kDa), and F4 (<1 kDa); (a) aspartic acid + asparagine, (b) glutamic acid + glutamine. Data (g of amino acid/ 100g of protein) are the average of three independent determinations ± standard deviation.

## Data Availability

The original contributions presented in this study are included in the article. Further inquiries can be directed to the corresponding authors.
